# “They feel like they have found a friend, and they are able to open-up and talk more”: A qualitative study on the role of Expert Clients and Health Promotion Officers in providing ART adherence support at Lighthouse Trust Clinics in Malawi

**DOI:** 10.64898/2026.08.05.26359835

**Published:** 2026-08-07

**Authors:** Odala Sande, Christine Kiruthu-Kamamia, Agness Thawani, Jacqueline Huwa, Gillian O’Bryan, Geldert Davie Chiwaya, Petros Tembo, Vivian Chipolombwe, Hannock Tweya, Lisa Orii, Caryl Feldacker

## Abstract

**Introduction:**

Expert clients (ECs) and Health Promotion Officers (HPOs) play a similar, and crucial, role in promoting client engagement and retention in antiretroviral therapy (ART) care. At Lighthouse Trust’s large public clinics in Lilongwe, Malawi, ECs/HPOs are HIV-positive clients who provide ongoing counseling to client peers during the first 12 months on ART. This study aims to explore the challenges EC/HPO face to gain insights for improving retention support services.

**Methods:**

Using a rapid qualitative study design, ten key informant interviews (KIIs) were conducted with ECs/HPOs at Lighthouse Trust’s two urban clinics. The interviews focused on retention challenges, strategies to increase client retention, and specific recommendations to improve client engagement.

**Results:**

Across the KIIs, ECs/HPOs expressed a strong sense of responsibility in supporting and motivating other people living with HIV. ECs/HPOs navigate complex client relationships by building trust and respecting cultural sensitivities. They consider themselves role models for ART adherence, disclosing their own HIV status to foster openness and encourage treatment continuity. While EC/HPOs tailor their conversations to clients’ needs, they remain disappointed by their inability to retain everyone in care. External factors, such as stigma and clients’ Socioeconomic status complicates their efforts. ECs/HPOs noted that ART clients who present as new clients for HIV testing to restart treatment instead of disclosing their treatment gap is particularly difficult to manage. To improve retention, ECs/HPOs recommended non-judgmental re-engagement strategies to encourage lost-to-follow-up clients to return to care, reduced documentation burdens, and promoted broadcasted ART retention messages on radio and television to support their work.

**Conclusions:**

The findings highlight the role of ECs/HPOs in building trust and adapting counseling approaches to support client engagement in ART care. Efforts are needed to encourage clients to return to ART services even after treatment gaps, with both EC/HPOs and the broader clinic community fostering a supportive care environment.

## Full Text Availability

The license terms selected by the author(s) for this preprint version do not permit archiving in PMC. The full text is available from the preprint server.

